# Real-Time Electrochemical Recording of Dopamine Release under Optogenetic Stimulation

**DOI:** 10.1371/journal.pone.0089293

**Published:** 2014-02-20

**Authors:** Wen-Tai Chiu, Che-Ming Lin, Tien-Chun Tsai, Chun-Wei Wu, Ching-Lin Tsai, Sheng-Hsiang Lin, Jia-Jin Jason Chen

**Affiliations:** 1 Department of Biomedical Engineering, National Cheng Kung University, Tainan, Taiwan; 2 Institute of Basic Medical Sciences, National Cheng Kung University, Tainan, Taiwan; 3 Medical Device Innovation Center, National Cheng Kung University, Tainan, Taiwan; 4 Department of Marine Biotechnology and Resources, National Sun Yat-Sen University, Kaohsiung, Taiwan; 5 Institute of Clinical Medicine, National Cheng Kung University, Tainan, Taiwan; 6 National Applied Research Laboratories, Taipei, Taiwan; National Institute of Health, United States of America

## Abstract

Dopaminergic PC12 cells can synthesize and release dopamine, providing a good cellular model for investigating dopamine regulation. Optogenetic stimulation of channelrhodopsin-2 provides high spatial and temporal precision for selective stimulation as a powerful neuromodulation tool for neuroscience studies. The aim of this study is to measure dopamine release from dopaminergic PC12 cells under optogenetic stimulation using electrochemical recording of self-assembled monolayers modified microelectrode with amperometric measurement in real time. The activation of PC12 cells under various optogenetic stimulation schemes are characterized by measuring single-cell Ca^2+^ imaging. After 10 seconds of optogenetic stimulation, the evoked intracellular Ca^2+^ level and dopamine current of channelrhodopsin-2-transfected PC12 cells were 1.6- and 3.5-fold higher than those of the control cells. The optogenetic stimulation effects on Ca^2+^ influx and dopamine release were 81% and 63% inhibition by using a Ca^2+^ channel antagonist Nifedipine. The results indicate that optogenetic stimulation can evoke voltage-gated Ca^2+^ channel-dependent dopamine exocytosis from PC12 cells in a cell specific, temporally precise and dose-dependent manner. This proposed dopamine recording system can be developed to be a good cell model for dopamine regulation and drug screening *in vitro*, or dopaminergic cell implantation therapy *in vivo* using optogenetic stimulation in a precise and convenient way.

## Introduction

Dopamine is an important catecholamine neurotransmitter implicated in physiological functions. Abnormal neurotransmission of dopamine can lead to numerous neurological disorders, such as Parkinson’s disease [1], [2], [3], [4]. Studies on dopamine regulation have shown that PC12 cells, a rat pheochromocytoma cell line, synthesize, release, and reuptake dopamine in a manner similar to that of dopaminergic neurons [5], [6]. Compared to brain neuron cultures or tissue slices, PC12 cell culture consists of a homogeneous dopamine-containing population, which had been widely used in dopaminergic cell investigations, including those on cell differentiation, neural protection, drug screening, and cell implantation therapies [7], [8], [9], [10], [11], [12], [13], [14]. Conventional approaches for regulating dopamine release in PC12 cells are pharmaceutical or electrical stimulation (ES) techniques, which have critical limitation on controlling dopamine release. Pharmaceutical stimulation such as KCl solution has poor temporal resolution and untargetable regulation of dopamine release from PC12 cells [15], [16]. Few studies have used ES for dopamine release because crosstalk and imprecise stimulation site control might result in controversial outcomes [17]. Among them, only some studies have demonstrated the inhibition effect of ES on dopamine release [17], [18]. These limitations restrict the application of dopamine release from PC12 cells as a precise cellular model for dopamine regulation.

Optogenetic stimulation approach that uses channelrhodopsin-2 (ChR2) has been developed for providing more precise and targeted stimulation effect on cells. ChR2-transfected neurons can be depolarized by exposure to ∼473 nm blue light in a very short duration (within 1 to 3 milliseconds), which immediately stops after the light is turned off. Because ChR2 expression can be targeted in specific types of cells, optogenetic stimulation allows potential therapeutic strategies to be investigated for neuroscience research and applications [19], [20], [21]. A lot of studies that utilized ChR2 for *in vivo* dopaminergic neuron stimulation successfully induced dopamine release in brain and conditioned animal behaviors [21], [22]. An optogenetic approach can thus be an effective tool for the dopaminergic regulation of cells, which could be an alternative model for the controlled release of dopamine in the Parkinson’s animal model.

Various techniques, including neuroimaging [23], microdialysis [24], and electrochemical [25] methods, have been developed for detecting dopamine levels. Compared to off-line neuroimaging and microdialysis methods, electrochemical detection provides higher temporal resolution in real time. To further improve the selectivity and sensitivity of dopamine sensing, various approaches using surface modifications on the sensing electrode have been developed [26]. The surface can be modified using self-assembled monolayers (SAMs), which provide a simple, convenient, and flexible method for functionalizing the chemical properties of the electrode-electrolyte interface [27], [28], [29]. Moreover, various nano-material-based modifications, such as those using gold nanoparticle (Au-NP) can provide larger effective areas for sensitivity enhancement to have low detection limit for dopamine recording [30], [31].

To better understand the optogenetic regulation of dopamine release from PC12 cells, the present study evaluates dopamine release from ChR2-tranfected PC12 cells using a surface-modified dopamine sensing electrode under various optogenetic stimulation schemes. Since intracellular Ca^2+^ is known to be essential for dopamine release from PC12 cells [32], studying the Ca^2+^ cascade effect in PC12 cells under optogenetic stimulation could provide in-depth information of the underlying mechanism. The results from Ca^2+^ signaling and real-time electrochemical monitoring of dopamine according to molecular imaging, we can delineate the Ca^2+^ dependency of dopamine release using blue light stimulation under different various Ca^2+^ buffers and voltage-gated Ca^2+^ channel (VGCC) antagonist. Combination of the ChR2-transfected PC12 cells, optogenetic stimulation and electrochemical recording could be a powerful model for dopamine research *in vitro*, which also has a high potential application to therapeutic treatment in animals.

## Materials and Methods

### Construction of the ChR2 Plasmid

The green algae *Chlamydomonas reinhardtii* (UTEX 2247) was purchased from the University of Texas Algal Collection (UTEX, USA). Total RNA extraction and reverse transcription by Trizol® Reagent (Invitrogen, USA) and SuperScript® III (Invitrogen, USA) were performed according to the manufacturer’s instructions. The cDNA encoding the first 315 amino acids of ChR2 (GenBank accession no. AF461397) was gendered by polymerase chain reaction amplified with the primers 5′-AACTGCAGATGGATTATGGAGGCGCCCT and 3′-GGATCCCGCTTGCCGGTGCCCTTGTTGA. The cDNA fragment of ChR2 was then inserted into cloning vector pEGFP-N1 (Clontech, USA) via the restriction sites of PstI and BamHI. The ChR2-EGFP plasmid was verified by DNA sequencing.

### Establishment of ChR2-transfected PC12 Cells

PC12 cells (BCRC 60048, Taiwan), a cell line derived from a pheochromocytoma of the rat adrenal medulla, were cultured at 37°C in RPMI 1640 medium supplemented with 15% horse serum, 5% fetal bovine serum, 1 mM sodium pyruvate, 25 units/mL penicillin, and 25 units/mL streptomycin. For plasmid DNA transfection, the ChR2-EGFP plasmid was transfected using Lipofetamine® 2000 (Invitrogen, USA). The ChR2-EGFP or the controlled EGFP stable expressing PC12 cells were selected by 600 mg/mL G418 over one month.

### Au-NP/SAM Modified Microelectrodes for Dopamine Release Recording

For dopamine release recording, SAM-modified microelectrodes with high sensitivity and selectivity to dopamine were designed and fabricated. The working electrode was prepared with platinum micro-wire (*Φ* = 75 µm, A–M Systems, USA) and inserted into a glass capillary which was drawn with a microelectrode puller. The end of the glass capillary was sealed with silicone rubber (Sylgard 184, Dow Corning, USA) and cured at 90°C for 12 hours. The tip of platinum microelectrode was cut by a scalpel, and the length of platinum micro-wire is extended ∼0.5 mm from the glass seal. For cleaning, the platinum plate (1.0 mm×1.2 mm) or microelectrode was cleaned for 30 minutes in each of ethanol and deionized water (18 MΩ·cm resistivity) and then immersed in 0.5 M H_2_SO_4_. Cyclic voltammetry (CV) was performed using an electrochemical analyzer (CHI 615C, CH Instruments, USA) from −0.2 V to 1.5 V (*vs.* Ag/AgCl electrode) until the CV curves were stable. In order to coat Au-NP, the platinum plate or microelectrode was immersed in 0.1 M HClO_4_ solution (Sigma, USA) containing 10 mM hydrogen tetrachloroaurate (HAuCl_4_, Sigma). A constant voltage of −0.5 V relative to the Ag/AgCl was applied. Au-NP were uniformly coated onto the surfaces of platinum plates and microelectrodes via electrodeposition. The surface morphologies of native platinum and Au-NP deposited platinum plates were evaluated by scanning electron microscopy (SEM, JSM-7001F, JEOL Ltd., Japan). To modify the self-assembled Au-NP monolayer, Au-NP deposited microelectrode was immersed in solutions of 5 mM mercaptopropionic acid (MPA, Sigma) dissolved in 95% ethanol for 15 minutes. The microelectrodes were finally washed copiously with ethanol to remove any non-binding thiols. Cells were stimulated by blue laser and recorded extracellular dopamine by electrode simultaneously at the sampling rate of 10 Hz.

### Optogenetic Stimulation of ChR2-transfected PC12 Cells

**Figure 1:**
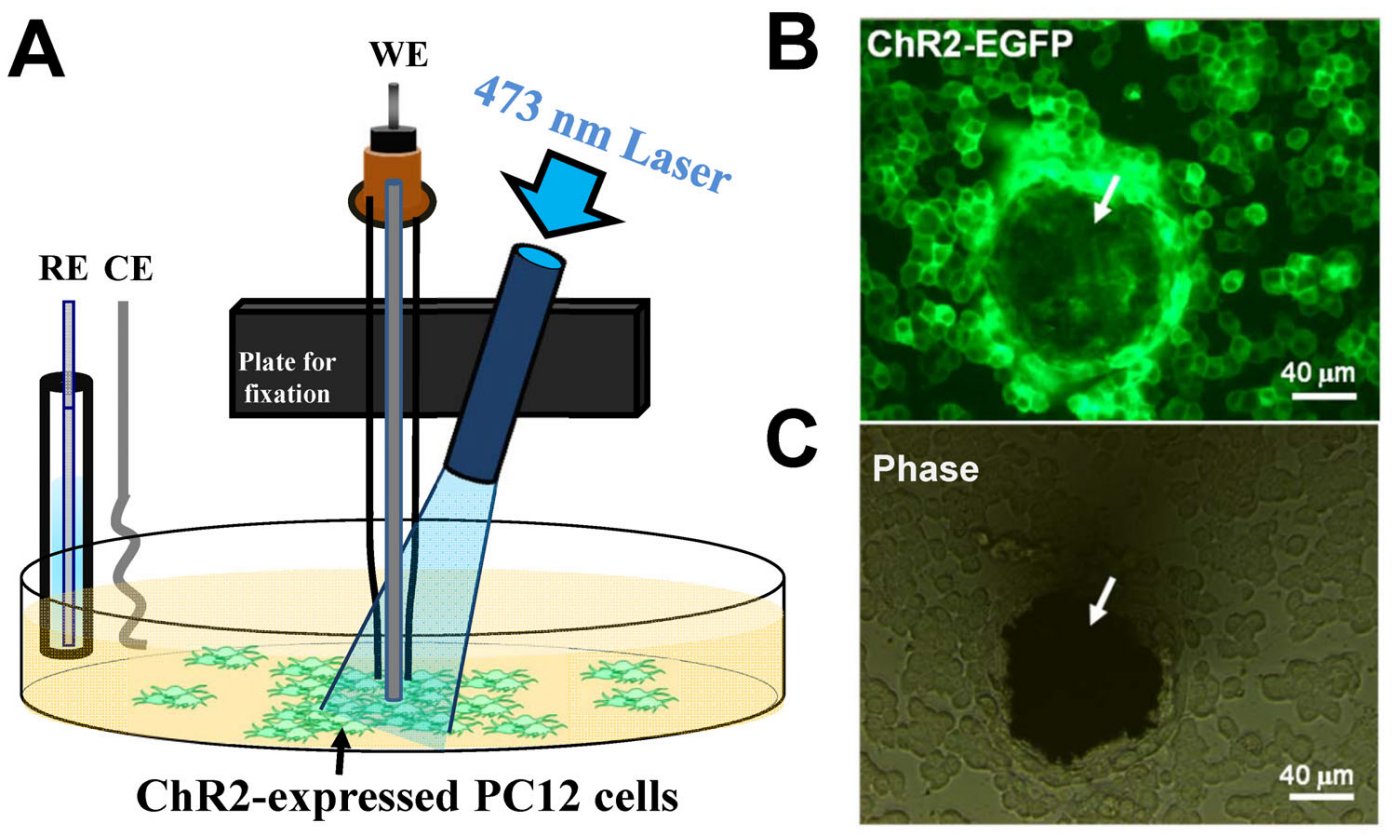
The *in vitro* real-time dopamine recording system. (A) Schematic diagram of working electrode (WE), counter electrode (CE), and reference electrode (RE) for dopamine sensing of ChR2-transfected PC12 cells under optogenetic stimulation at 473 nm. (B) Fluorescence and (C) phase-contrast images of ChR2-EGFP-expressing PC12 cells and electrode (arrows) under wide-field fluorescence microscope.

For all optogenetic stimulation experiments, a 473-nm fiber-coupled diode-pumped solid-state (DPSS) laser (MBL-III-473-50 mW, Laser 2000 Ltd., UK) with TTL modulation was used. The output power of the laser was adjusted to 10 mW/mm^2^ and confirmed by an optical power meter (Ophir Optronics Ltd., Israel). The tip of the 400-µm fiber was fixed on a micromanipulator for guiding the blue light laser to the targeted cells. 1×10^6^ cells/mL of ChR2-EGFP or EGFP stable expressing PC12 cells were seeded on a 25 µg/mL poly-D-lysine (PDL)-coated 6-well plate (*NUNC, Denmark*) with complete growth medium before optogenetic stimulation experiments. After culture for 24 hours at 37°C, the medium was replaced by 1.8 mM Ca^2+^-contained phosphate-buffered saline (PBS), Ca^2+^-free PBS, or 10 µM Nifedipine (Sigma, USA) in Ca^2+^-containing PBS. For optogenetic stimulation and dopamine sensing, the optical fiber and electrodes were fixed on the micromanipulator and aimed at the targeted cell as closely as possible, as shown in Figure 1. The reference electrode and counting electrode were immersed in medium in dish. The working electrode for dopamine sensing and laser fiber were fixed together and manipulated to close to cells. Each experiment consisted of a 20-second baseline recording, 10-second blue laser stimulation, and at least a 3-minute recovery time before the subsequent stimulation. The optogenetic stimulations were validated at stimulation frequencies of 10, 20, and 30 Hz at pulse duration of 10, 20, and 30 ms. The illumination was fixed at 30 Hz and 30 ms in this study after observing the effects of varied stimulation parameters.

### Recording of Intracellular Ca^2+^ Levels

Intracellular Ca^2+^ levels were measured using the ratiomatric fluorescent dye fura-2/AM (Invitrogen, USA) under a single-cell fluorimeter (TILL Photonics, Germany). 1×10^6^ PC12 cells/mL were seeded on a 25 µg/mL PDL-coated glass bottom dish and cultured in complete growth medium at 37°C for 24 hours. Cells were loaded with 5 µM fura-2/AM in PBS at 26°C for 1 hour. After loading, cells were washed three times with PBS and recovered for 30 minutes. Dishes were then placed on the stage of the single-cell fluorimeter for Ca^2+^ measurement. The ratiometric Ca^2+^ indicator, fluorophore fura-2/AM, was excited by fast switched 340 nm and 380 nm wavelengths for 50 ms, and emissions were the same at 510 nm. The duration between fast switched 340 nm and 380 nm wavelengths is 2 ms, and the illumination intensity is 10 mW. The relative Ca^2+^ level was calculated from the ratio of the emission intensities with excitations of 340 nm and 380 nm (ratio 340/380). Intracellular fura-2 fluorescence was sequentially imaged and the mean fluorescence intensity of individual PC12 cells was quantitatively analyzed by TILLvisION software of the TILL Photonics system (TILL Photonics, Germany). The Ca^2+^ imaging techniques were applied to ChR2-transfected PC12 cells with various optogenentic stimulation schemes.

### Statistical Analysis

All data were represented as mean ± SEM (standard error of the mean) in each experiment. Student’s *t*-test and ANOVAs were used for statistical analyses. A *p* value < 0.05 is considered as significantly different.

## Results

### Dopamine Sensing Using Au-NP/SAM Modified Microelectrodes

**Figure 2:**
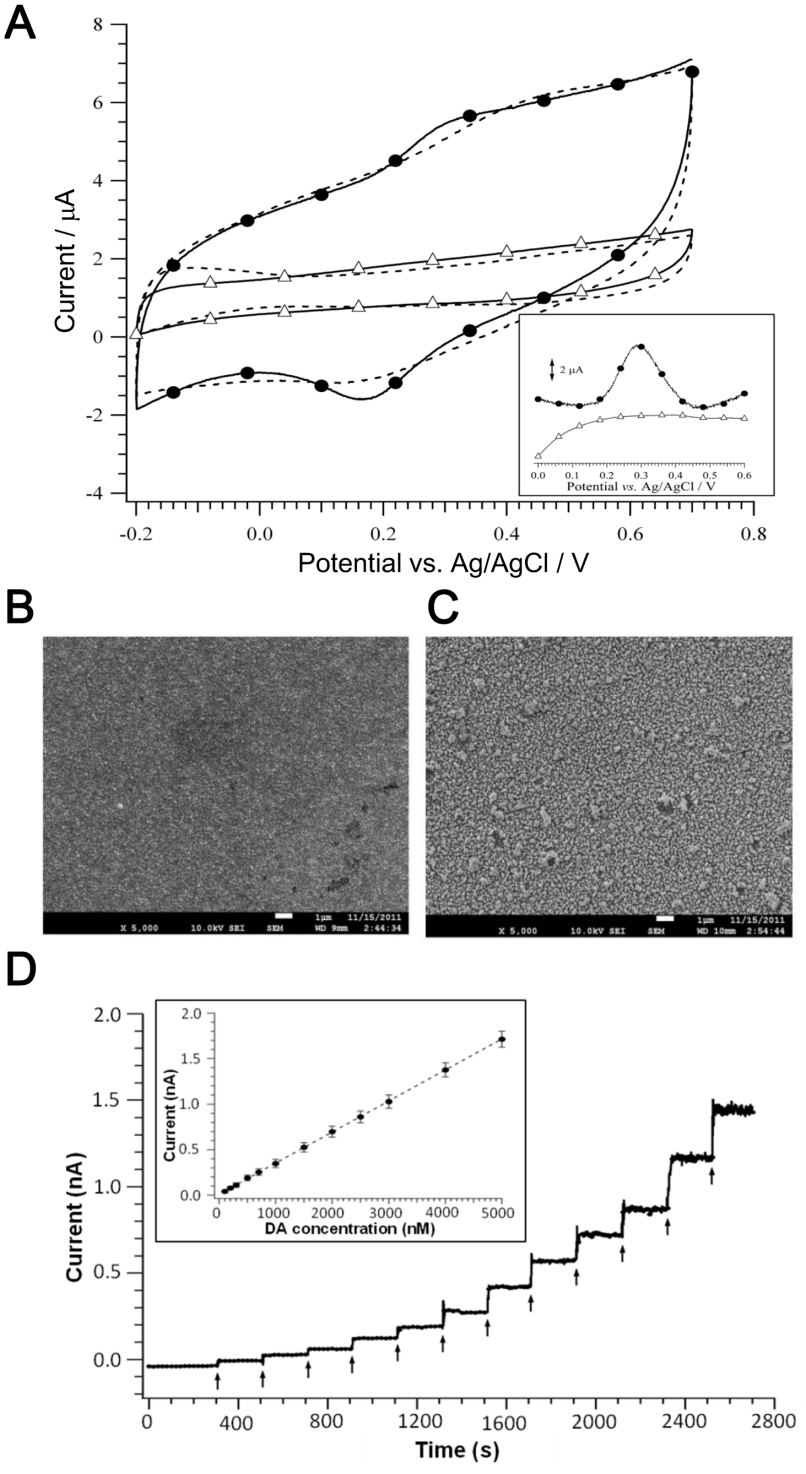
Characterization of Au-NP/SAM modified electrodes. (A) CV curves of 10 µM dopamine recorded by native (-Δ-) and Au-NP/SAM (-•-) platinum microelectrodes at a scan rate of 10 V s^−1^. The inserted picture represents the CV curve after background subtraction. SEM images showing the surface morphologies of (B) native and (C) Au-NP/SAM platinum electrodes. (D) Amperometric *i-t* curves of Au-NP/SAM microelectrodes recorded in PBS for dopamine calibration at a scan potential of 0.25 V (*vs.* Ag/AgCl).

Figure 2A shows that cyclic voltammograms (CV) of native platinum (open triangles) and Au-NP/SAM (open circles) modified microelectrodes with 10 µM dopamine. The CV scans were applied from −0.2 V to 0.7 V in PBS containing dopamine at a rate of 10 V s^−1^. A pair of well-defined peaks for dopamine redox can be observed at Au-NP deposited microelectrode, with an oxidative peak potential at 0.25 V (*vs.* Ag/AgCl) and corresponding reductive peak potential at around 0.16 V. In contrast, these peaks of dopamine redox were not obvious on the CV curves of native platinum microelectrodes. In Figures 2B and 2C, the surface morphologies of native platinum and Au-NP deposited platinum substrates were obtained by SEM. The SEM images show that the Au-NP uniformly covered the entire surface and formed a well-ordered distribution on the substrate under electrodeposition. Figure 2D exhibits a step-like increment of current corresponding to the successive additions of dopamine solution. The insert in Figure 2D reveals the correlation between the responsive current and the dopamine concentration at Au-NP/SAM modified microelectrode. The linear working range of dopamine is obtained from 0.1 µM to 5 µM at this microelectrode. The linear regression has a correlation coefficient (*R* ^2^) of 0.999 and a detection limit of 7 nM at a signal-to-noise ratio of 3.

### Dopamine Release from ChR2-activated PC12 Cells

**Figure 3:**
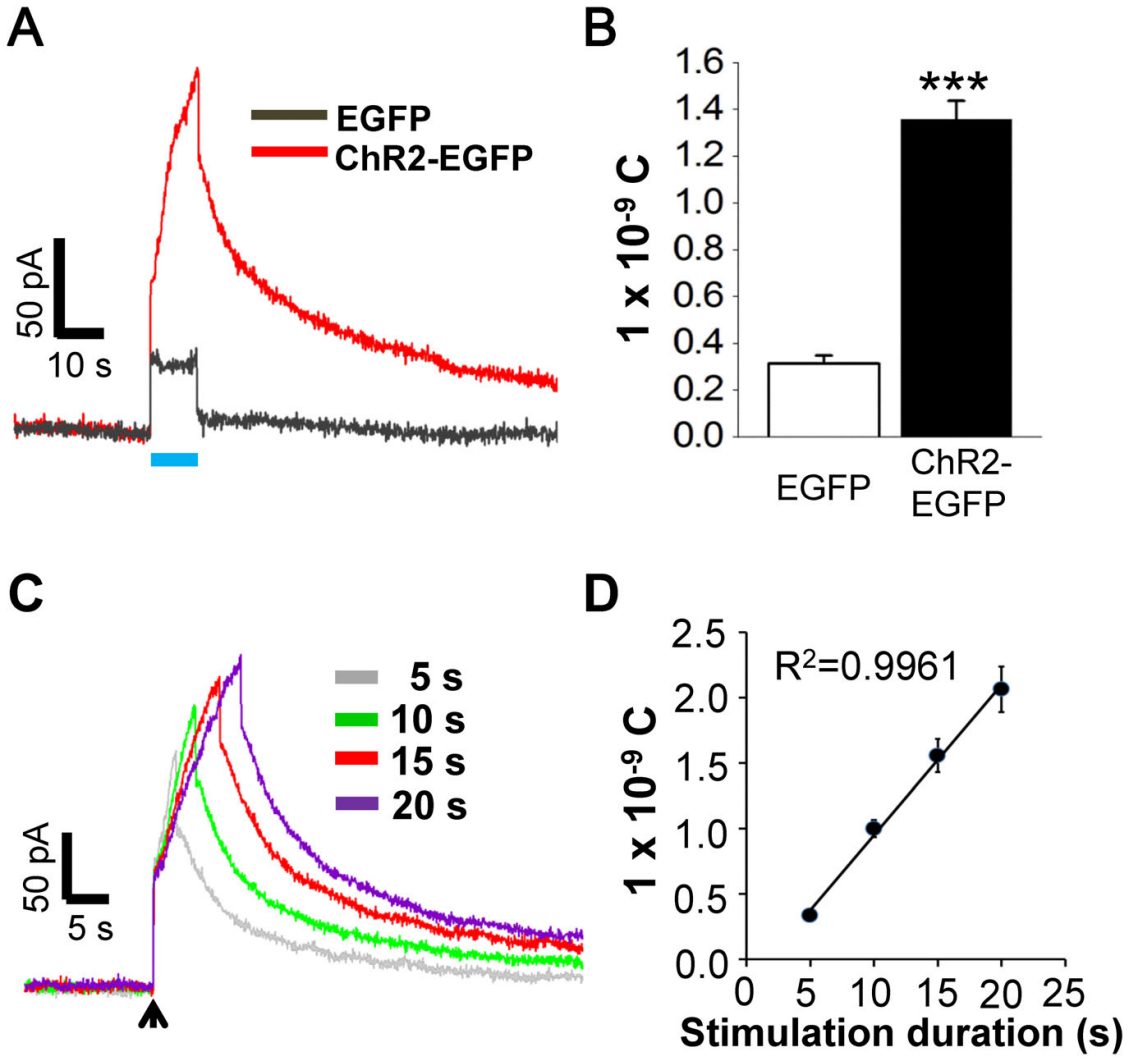
Dopamine release by optogenetic stimulation. (A) Extracellular dopamine level recording with blue light illumination for 10 seconds on EGFP- and ChR2-EGFP-expressing PC12 cells. (C) ChR2-mediated dopamine release under different illumination durations, from 5 seconds to 20 seconds. Arrow indicates the start of illumination. (A) and (C) are dopamine signals in current vs. time. (B) and (D) are integrations of evoked currents with time during blue light illumination. Each column represents mean ± SEM from five independent experiments. ***, *P*<0.001.

The controlled EGFP- or ChR2-EGFP-expressing PC12 cells were cultured in dish for dopamine release monitoring. After the medium was replaced with PBS, the sensing electrode recorded the extracellular concentration of dopamine change upon 10 seconds of blue light illumination. As shown in Figures 3A and 3B, upon optical illumination, the photoelectrical artifact of 0.31±0.03×10^−9^ C can be observed in the controlled EGFP-expressing cells. In contrast, optogenetic stimulation evoked immediate elevations of dopamine signals about 1.36±0.08×10^−9^ C in ChR2-EGFP-expressing cells, which is significantly higher than that of the control (t_(8)_ = −11.91, *p*<0.001; Student’s *t*-test). After termination of the blue light, the dopamine signals slowly decreased and returned towards the base values. Then, four durations (5, 10, 15, and 20 seconds) of blue light illumination were used to investigate the influence of stimulation duration on dopamine release from ChR2-expressing PC12 cells. As shown in Figures 3C and 3D, the detected dopamine levels gradually and linearly (*R* ^2^ = 0.996) increased from 0.33±0.02, 0.99±0.03, 1.55±0.07 to 2.06±0.10×10^−9^ C for 5, 10, 15, and 20 seconds of stimulation duration, respectively. The photoelectrical artifacts were removed by direct subtraction.

### Characterization of the Role of Ca^2+^ during ChR2-evoked Dopamine Release

**Figure 4:**
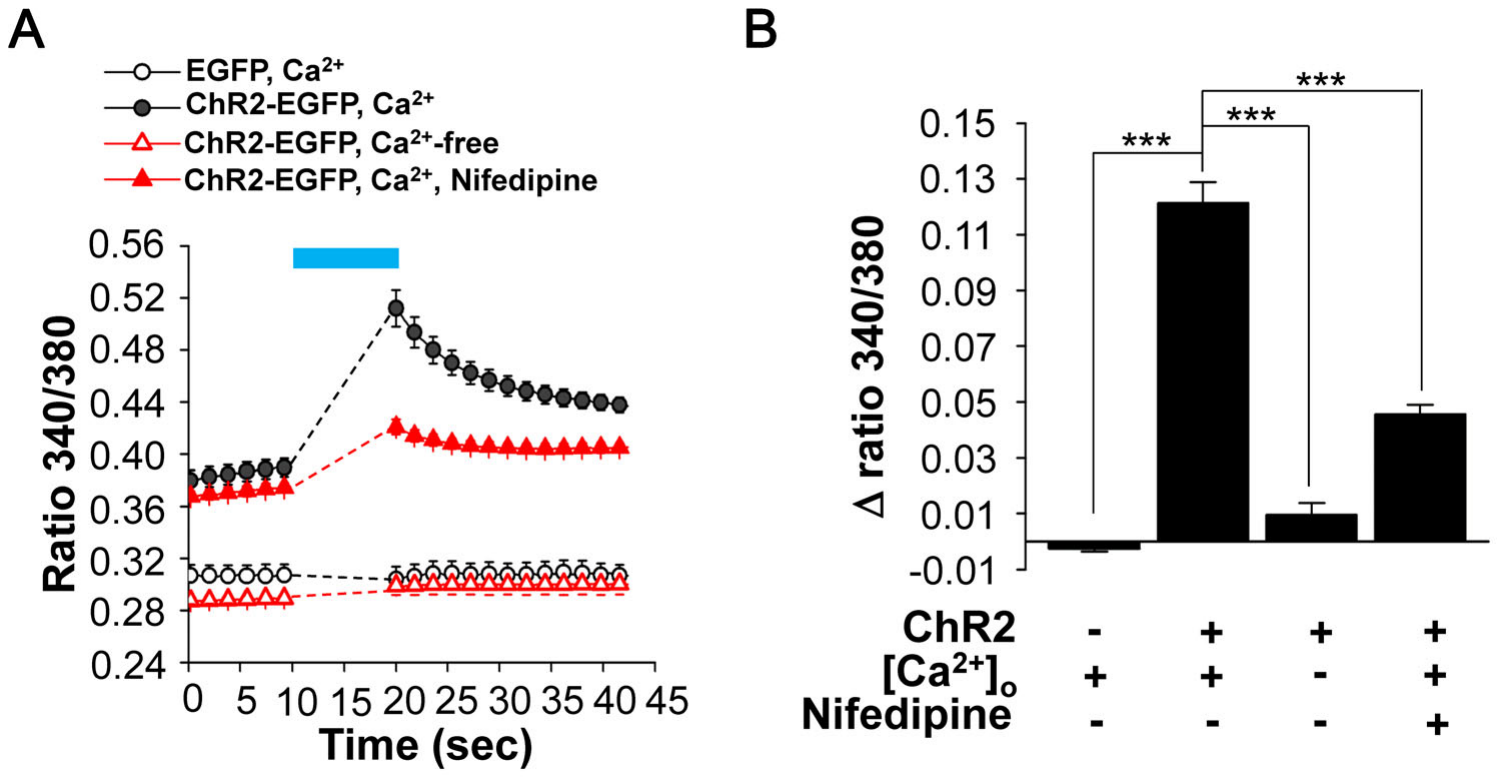
Extracellular calcium influx during optogenetic stimulation. (A) Relative intracellular Ca^2+^ levels of ratiometric fura-2 fluorescence intensity (ratio 340/380) for EGFP- and ChR2-EGFP-expressing PC12 cells in 1.8 mM Ca^2+^ buffer with or without 10 µM Nifedipine, or Ca^2+^-free buffer under illumination of blue light for 10 seconds. The Ca^2+^ signals during light illumination that could not be read out are depicted as dotted lines. (B) The changes of relative intracellular Ca^2+^ levels (mean ± SEM) by the fura-2 ratiometric imaging (Δ ratio 340/380) from at least 50 cells of four independent experiments. ***, *P*<0.001.

It is well known that L-type VGCC plays an important role on dopamine release in PC12 cells. However, the optogenetic effect on the Ca^2+^ cascade is not clear. To investigate the optogenetic effect on the Ca^2+^ cascade, optogenetic stimulation were applied to PC12 cells in Ca^2+^-free buffer, 1.8 mM Ca^2+^ buffer and L-type VGCC antagonist Nifedipine-containing buffer (10 µM Nifedipine in 1.8 mM Ca^2+^ buffer). Relative intracellular Ca^2+^ levels are presented as the ratiometric fura-2 fluorescence intensity (ratio 340/380) by single-cell fluorimeter (two-way ANOVA of ChR2-EGFP/Ca^2+^ vs. EGFP/Ca^2+^ (*p*<0.001), ChR2-EGFP/Ca^2+^ vs. ChR2-EGFP/Ca^2+^-free (*p*<0.001), ChR2-EGFP/Ca^2+^ vs. ChR2-EGFP/Ca^2+^/Nifedipine (*p*<0.01); ChR2-EGFP/Ca^2+^/Nifedipine vs. ChR2-EGFP/Ca^2+^-free (*p*<0.001), ChR2-EGFP/Ca^2+^/Nifedipine vs. EGFP/Ca^2+^ (*p*<0.001) with Bonferroni’s post test; Figure 4A). As shown in Figure 4B, the intracellular Ca^2+^ change of ChR2-EGFP-expressing PC12 cells (Δ ratio 340/380 = 0.121±0.007) was significantly higher than that of the controlled EGFP-expressing PC12 cells (Δ ratio 340/380 = −0.002±0.001) after 10 seconds of blue light illumination. In contrast, the intracellular Ca^2+^ change was inhibited (Δ ratio 340/380 = 0.009±0.004) when ChR2-EGFP-expressing PC12 cells were immersed in Ca^2+^-free buffer. Similarly, Nifedipine significantly inhibited the change of Ca^2+^ level (Δ ratio 340/380 = 0.045±0.003) upon optogenetic stimulation (F_(3,12)_ = 146.8, *p*<0.001; one-way ANOVA with Tukey’s multiple comparisons test).

**Figure 5:**
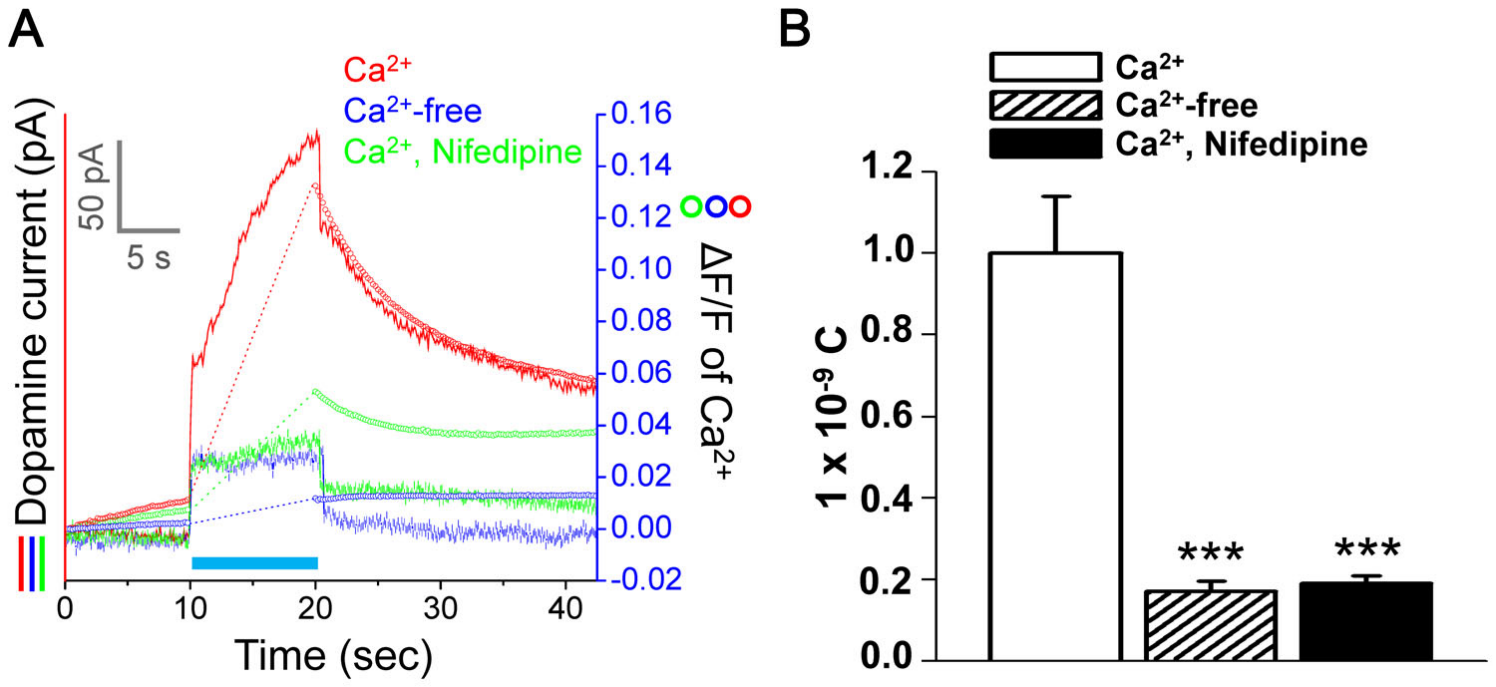
Effect of calcium influx on ChR2-mediated dopamine release. (A) Simultaneous optical stimulation, dopamine current recording and Ca^2+^ imaging. Blue light illumination on ChR2-EGFP-expressing cells in controlled 1.8 mM Ca^2+^ buffer without or with 10 µM Nifedipine, and Ca^2+^-free buffer. The light-related artifact is not removed. (B) Significant inhibition in dopamine current during blue light illumination in Ca^2+^-free and Ca^2+^ buffer with Nifedipine. Each column represents mean ± SEM from five independent experiments. ***P<0.001.

It is known that that the L-type VGCC antagonist Nifedipine could block Ca^2+^ influx during optogenetic stimulation from Figure 4. As shown in Figure 5A, we further recorded the extracellular dopamine during optogenetic stimulation from ChR2-expressing cells under normal, Ca^2+^-free buffer (0.17±0.02×10^−9^ C) and Nifedipine/1.8 mM Ca^2+^ (0.09±0.01×10^−9^ C) (two-way ANOVA of Ca^2+^ vs. Ca^2+^-free (*p*<0.001), Ca^2+^ vs. Ca^2+^/Nifedipine (*p*<0.001), Ca^2+^-free vs. Ca^2+^/Nifedipine (*p*>0.05, non-significant) with Bonferroni’s post test). The dopamine currents were significantly inhibited in both Ca^2+^-free buffer (0.17±0.02×10^−9^ C) and Nifedipine/1.8 mM Ca^2+^ (0.19±0.01×10^−9^ C) conditions in comparison to that for the 1.8 mM Ca^2+^ buffer (1.00±0.13×10^−9^ C) condition with the artifacts removed (F_(2,12)_ = 33.52, *p*<0.001; one-way ANOVA with Tukey’s multiple comparisons test; Figure 5B).

### Dopamine Release and Ca^2+^ Influx Under Various Optogenetic Stimulation Schemes

**Figure 6:**
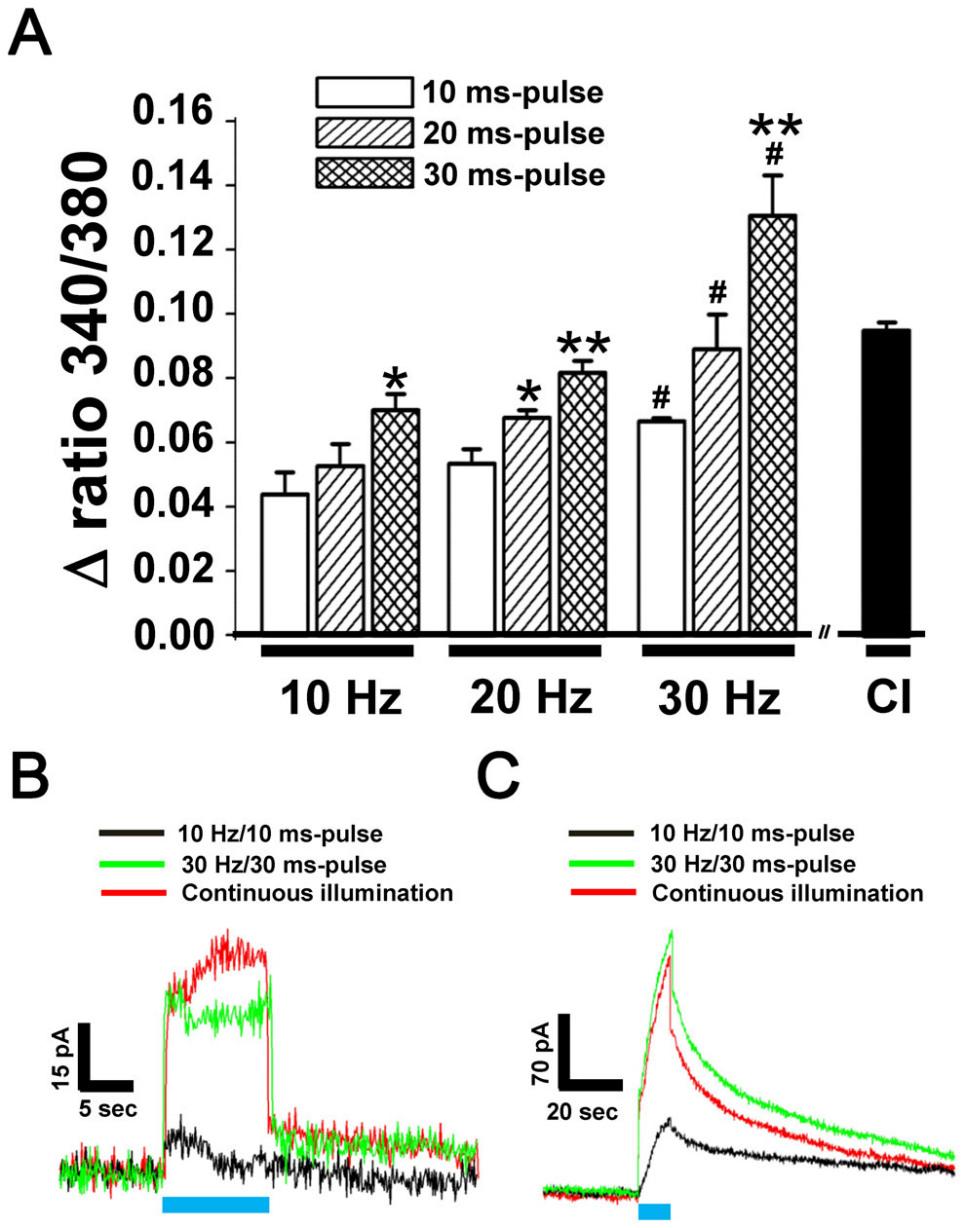
Effect of stimulation parameters of blue light on calcium influx and dopamine release. Optogenetic stimulation at frequencies of 10, 20, and 30(CI) of blue laser light were applied for 10 seconds. (A) Changes of relative intracellular Ca^2+^ levels by the fura-2 ratiometric imaging (Δ ratio 340/380). Each column represents mean ± SEM from at least 60 cells of three independent experiments. *: significant difference within the same group; #: significant difference between groups. # and *, *P*<0.05; **, *P*<0.01. (B) Background current of stimulatory artifacts and (C) dopamine release under 10 Hz/10 ms, 30 Hz/30 ms, and CI of blue light.

To examine the effect of different stimulation schemes including stimulation frequency and pulse duration on the dopamine release and influx of Ca^2+^ from ChR2-EGFP-expressing PC12 cells. For comparison, continuous illumination (CI) and stimulatory pulses of blue light with frequencies of 10, 20, and 30 Hz and durations of 10, 20, and 30 ms were applied to cells for 10 seconds. Figure 6A shows that the Ca^2+^ levels of PC12 cells increased with the increase of stimulatory frequency from 10 Hz to 30 Hz as well as with the increase of stimulation pulse duration. The highest elevation of Ca^2+^ (Δ ratio 340/380 = 0.130±0.020) occurred at stimulation pulses of 30 Hz and 30 ms which is about 3-fold of 10 Hz/10 ms stimulation pulse (Δ ratio 340/380 = 0.043±0.006). CI led to lower Ca^2+^ elevation (Δ ratio 340/380 = 0.095±0.004) compared to the 30 Hz/30 ms illumination (10 Hz/30 ms vs. 10 Hz/10 ms, t_(4)_ = 3.135, *p*<0.035; 20 Hz/30 ms vs. 20 Hz/10 ms, t_(4)_ = 4.855, *p*<0.0083; 20 Hz/20 ms vs. 20 Hz/10 ms, t_(4)_ = 2.854, *p*<0.0462; 30 Hz/30 ms vs. 30 Hz/10 ms, t_(4)_ = 5.048, *p*<0.0072; 30 Hz/10 ms vs. 10 Hz/10 ms, t_(4)_ = 3.264, *p*<0.031; 30 Hz/20 ms vs. 10 Hz/20 ms, t_(4)_ = 2.835, *p*<0.0471; 30 Hz/30 ms vs. 10 Hz/30 ms, t_(4)_ = 4.467, *p*<0.0111; 30 Hz/10 ms vs. 20 Hz/10 ms, t_(4)_ = 2.858, *p*<0.046; 30 Hz/30 ms vs. 20 Hz/30 ms, t_(4)_ = 3.704, *p*<0.0.0208 with Student’s *t*-test).

The dopamine release of controlled EGFP- and ChR2-EGFP-expressing cells under various stimulation schemes was also observed. The photoelectrical artifacts of detected currents of EGFP-expressing PC12 cells under various stimulation schemes of blue light illumination were examined. The results are shown in Figure 6B. As expected, CI (0.368±0.026×10^−9^ C) and the 30 Hz/30 ms pulse (0.314±0.034×10^−9^ C) of blue light illumination evoked higher background dopamine currents compared to the 10 Hz/10 ms pulse (0.007±0.011×10^−9^ C). Figure 6C shows the dopamine signals from ChR2-expressing PC12 cells under various schemes of blue light stimulation. The dopamine current from 10 Hz/10 ms stimulation (0.319±0.029×10^−9^ C) was far lower than those of the 30 Hz/30 ms stimulation (1.215±0.135×10^−9^ C) and CI (1.006±0.128×10^−9^ C) with artifacts removed.

## Discussion

It is important to develop a delicate tool for effective control of dynamic process of dopamine, release or reuptake, from cells and real-time monitoring of extracellular dopamine levels for neuroscience investigation or clinical application. The developed microelectrodes at a diameter of 0.1 µm was enhanced with Au-NP sensing surface area for high sensitivity of detection limit of 7 nM for monitoring dopamine release from cells. Previous studies recorded the elevation of environmental dopamine levels at 10 nM/min/5×10^4^ PC12 cells in 100 µL of medium under long period (5 hours) of high K^+^ stimulation [33]. In contrast, our highly sensitive electrodes were placed at the vicinity of stimulated PC12 cells for immediate (on the order of seconds) sensing of dopamine release. Our approach combines optogenetic stimulation and amperometry of Au-NP/SAM microelectrode is capable of monitoring the rapid dopamine release from ChR2-expressing PC12 cells compared to the common use of high K^+^ buffer (60 mM) for PC12 cell membrane depolarization. Our approach overcomes the disadvantages of a long diffusion time and the continuous replacement of K^+^ buffer, which is infeasible for studies *in vivo*. The present study validated that the evoked dopamine signals in ChR2-EGFP-expressing cells are significantly higher than those of EGFP-expressing cells (p<0.001, unpaired *t*-test). ChR2-mediated Ca^2+^ influx can active depolarized dopaminergic PC12 cells. An immediate dopamine release signal of around ∼30 pA (artifact-removed) can be recorded within 100 ms. On the other hand, the evoked dopamine signals can reach to 155 pA after 10 seconds of optogenetic stimulation. The signal stops rising immediately and slowly returns to the basal line in around 3 minutes upon dopamine reuptake and diffusion after turn off blue light.

Moreover, the dopamine signal was elevated about 3.5-fold higher than those of cells without optogenetic stimulation, whereas the conventional high K^+^ stimulation passive depolarized cells only elevated 1.8-fold of dopamine level in 20 minutes from 5×10^5^ cells/well of PC12 cells [34]. The proposed optogenetic stimulation with sensitive dopamine sensing microelectrode approach could provide immediate response of controlled dopamine release, which is a powerful model for investigate the effects of optogenetic stimulation on dopamine regulation of dopaminergic cells.

Previous studies have demonstrated that optogenetic stimulation can induce cell membrane depolarization of PC12 cells. The relation between blue light intensity and evoked potential has been examined [35]. Moreover, Chang *et* *al.* measured Ca^2+^ dynamics in PC12 cells upon optogenetic stimulation by fluorescent indicators [36]. Based on these results, the present study further examined the optogenetic effects on the voltage-dependent Ca^2+^ influx and the relationship to dopamine release. Single-cell Ca^2+^ measurement of fura-2/AM was used to characterize the optogenetic stimulation effect on the Ca^2+^ cascade in PC12 cells. In our findings, blue light did not affect the intracellular Ca^2+^ level in PC12 cells without ChR2 expression. In contrast, the Ca^2+^ level was significantly elevated by activation of ChR2 upon blue light illumination. The 340/380 ratio was elevated about 1.6-fold of control upon 10 seconds of optogenetic stimulation. However, the elevation immediately stopped and slowly returned to the basal level after the blue light was turned off. These results show the specific effect and temporal precision of optogenetic stimulation. Moreover, our findings indicate that PC12 cells with ChR2-EGFP expression had a higher basal intracellular Ca^2+^ level than that of EGFP-expressing cells, which might be caused by the self-leakage of ChR2 itself and/or activation of ChR2 by the ultraviolet light (340 nm and 380 nm) used for Ca^2+^ recording. Ca^2+^-free buffer and the L-type VGCC antagonist, Nifedipine, were used to explore the effect of optogenetic stimulation on extracellular Ca^2+^ influx. Nifedipine is a kind of L-type VGCC inhibitor, which can be a blocker in Ca^2+^-dependent dopamine release. Abu-Raya *et* *al.* showed that Nifedipine can block about 25% of intracellular Ca^2+^ elevation upon high K^+^ stimulation [34]. Our results show that Nifedipine significantly inhibited about 63% of Ca^2+^ elevation induced by optogenetic stimulation. Additionally, the intracellular Ca^2+^ level barely changed when cells were immersed in Ca^2+^-free buffer. These results indicate that extracellular Ca^2+^ influx is the major source for cytosolic Ca^2+^ elevation during optogenetic stimulation. Then, the L-type VGCC plays an essential role in optogenetic stimulation-evoked Ca^2+^ influx. This phenomenon can be explained as ChR2-mediated extracellular Ca^2+^ influx slightly depolarized PC12 cells, which turns to activate L-type VGCC then following large Ca^2+^ influx. Compared to those using high K^+^ buffer, the optogenetic approach is more controllable and can be easily manipulated using Ca^2+^-free buffer or L-type VGCC blocker.

The effects of Ca^2+^-free buffer and Nifedipine on the modulation of dopamine release were examined after the cascade of Ca^2+^ influx was characterized. Our results in Figure 5 indicate that 83% (Ca^2+^-free PBS) and 81% (Nifedipine in 1.8 mM Ca^2+^ PBS) inhibition of dopamine signals were found in comparison to that of the controlled group (1.8 mM Ca^2+^-containing PBS). The high correlation between Ca^2+^ influx and dopamine release indicates that L-type VGCC might play a major role in dopamine release from PC12 during optogenetic stimulation. As shown in Figure 6, the intracellular Ca^2+^ elevation by blue light is in a frequency- and time-dependent manner. The effects of illumination parameters were similar in Ca^2+^ influx and dopamine release experiments. However, a higher power-level of blue light caused stronger artifacts in the dopamine signal as Figure 6B, which should be removed for accurate estimation. Either duration or averaged power of the laser is an important component in increasing the artifact current. Both parameters are related to the total energy exerted on the dopamine-sensitive electrode. So, the light stimulation-induced thermal effects on the electrode might be the major factor.

The problem and limitation on this study is the overlap between excitation wavelengths for Ca^2+^ imaging (340 nm and 380 nm) and for activation of ChR2 (325–550 nm). It may cause a large portion of ChR2 has already activated by 340 nm and 380 nm before a start of 473 nm pulses. Also, 380 nm is more efficient in activating ChR2 than 340 nm, causing a biased readout on the 340/380 ratio. In order to obtain continuous Ca^2+^ measurement during 473 nm illumination for ChR2 excitation, the potential solutions to overcome this problem by using red Ca^2+^ indicator with ChR2, green Ca^2+^ indicator with red-shifted ChR2, or using the bioluminescent Ca^2+^ indicator Nano-lantern [37] with ChR2 to avoid spectrum overlapping. To date, many red Ca^2+^ indicators are available with the apply on ChR2 stimulation, including synthetic dyes (e.g., Calcium Orange™ and CaTM-2/AM) and protein-based red Ca^2+^ sensors (e.g., R-GECOs, RCaMPs, O-GECO and CAR-GECO) [38], [39], [40]. Furthermore, optogenetic stimulation can be performed within the “dead time” of the charge-coupled device camera to apply simultaneous optical stimulation and fluorescence imaging [36].

Our results demonstrated that optogenetic stimulation could evoke and precisely control dopamine signals from PC12 cells. The extracellular dopamine signals can be evoked immediately within 100 ms when the ChR2-expressing cells were illuminated by blue light and slowly returned to the baseline after the stimulation was turned off. Optogenetic stimulation could be a powerful tool for activation of PC12 cells and controlling of dopamine release at high temporal resolution, easy manipulation and less side effects. In addition, the L-type VGCC on membrane might play a key role in the evoked Ca^2+^ influx and dopamine release under optogenetic stimulation. Furthermore, varied stimulation schemes to modulate ChR2 activation of PC12 cells have been examined. The 30 Hz with 30 ms pulse duration evoked more dopamine release than CI stimulation. The cationic channel ChR2 with sequenced activation and inactivation cycles based on its channel nature. So, the CI stimulation didn’t evoke more dopamine release compared to the 30 Hz with 30 ms pulse duration might due to it unreached the maximal activation of ChR2. The traditional stimulation approaches for dopamine release always limited the therapeutic potential of their studies by used of pharmaceutical or electrical stimulations. Based on our result, novel optogenetic stimulation approach would open powerful modulation scheme for further clinical application and investigation of dopamine regulation**.**
